# Interaction of cytokeratin 19 head domain and HER2 in the cytoplasm leads to activation of HER2-Erk pathway

**DOI:** 10.1038/srep39557

**Published:** 2016-12-23

**Authors:** Tomoaki Ohtsuka, Masakiyo Sakaguchi, Hiromasa Yamamoto, Shuta Tomida, Katsuyoshi Takata, Kazuhiko Shien, Shinsuke Hashida, Tomoko Miyata-Takata, Mototsugu Watanabe, Ken Suzawa, Junichi Soh, Chen Youyi, Hiroki Sato, Kei Namba, Hidejiro Torigoe, Kazunori Tsukuda, Tadashi Yoshino, Shinichiro Miyoshi, Shinichi Toyooka

**Affiliations:** 1 Departments of Thoracic, Breast and Endocrinological Surgery, Okayama University Graduate School of Medicine, Dentistry and Pharmaceutical Sciences, 2-5-1 Shikata-cho, Kita-ku, Okayama 700-8558, Japan; 2 Department of Cell Biology, Okayama University Graduate School of Medicine, Dentistry and Pharmaceutical Sciences, 2-5-1 Shikata-cho, Kita-ku, Okayama 700-8558, Japan; 3 Biobank, Okayama University Graduate School of Medicine, Dentistry and Pharmaceutical Sciences, 2-5-1 Shikata-cho, Kita-ku, Okayama 700-8558, Japan; 4 Department of Pathology, Okayama University Graduate School of Medicine, Dentistry and Pharmaceutical Sciences, 2-5-1 Shikata-cho, Kita-ku, Okayama 700-8558, Japan; 5 Department of Clinical Genomic Medicine, Okayama University Graduate School of Medicine, Dentistry and Pharmaceutical Sciences, 2-5-1 Shikata-cho, Kita-ku, Okayama 700-8558, Japan

## Abstract

HER2 is a receptor tyrosine kinase and its upregulation via activating mutations or amplification has been identified in some malignant tumors, including lung cancers. Because HER2 can be a therapeutic target in HER2-driven malignancies, it is important to understand the molecular mechanisms of HER2 activation. In the current study, we identified that cytokeratin 19 (KRT19) binds to HER2 at the inside face of plasma membrane. HER2 and KRT19, which were concurrently introduced to a human embryonic kidney 293 T cells, revealed an association with each other and resulted in phosphorylation of HER2 with the subsequent activation of a downstream Erk-associated pathway. A binding assay revealed that both the NH2-terminal head domain of KRT19 and the COOH-terminal domain of HER2 were essential for their binding. To investigate the impact of the interaction between HER2 and KRT19 in lung cancer, we examined their expressions and localizations in lung cancers. We found that KRT19 was highly expressed in HER2-positive lung cancer cells, and KRT19 and HER2 were co-localized at the cell membrane. In conclusion, we found that KRT19 intracellularly binds to HER2, playing a critical role in HER2 activation.

HER2 is a human epidermal growth factor receptor (HER) family protein and is known to be expressed in many malignancies. The overexpression of HER2 is reportedly observed in about 30% of non-small cell lung cancer (NSCLC)^1^,^2^,^3^,^4^. Mutations in the tyrosine kinase domain of *HER2* have been detected in 2–4% of lung adenocarcinomas^5^,^6^,^7^. Considering these findings, uncovering molecular interaction involved in HER2 signaling is critical to understand HER2 related oncogenesis and to develop the new treatments for HER2-alterated malignancies. Recently, we found the novel functional mutations in the transmembrane domain (TD) (codons 659 and 660) of *HER2*^8^. These *HER2* mutations are considered to be the oncogenic mutations in certain histological types of lung cancers^9^,^10^,^11^. These mutant sites in the TD are known to important for dimerization of HER2 and we speculated that the partners of dimerization of the TD mutant HER2 may be different from those of wild type HER2. Thus, we investigated the possible partners of TD mutant HER2. In the course of identifying novel partner receptor for TD mutant HER2, we found that cytokeratin 19 (KRT19) is bind to wild type HER2 in A549 lung cancer cell line. KRT19, which is a member of the keratin intermediate filament family of proteins, is well known to be generally overexpressed in various cancers^12^,^13^,^14^,^15^,^16^,^17^, and its fragment known as CYFRA has been shown to be a tumor marker in some subsets of lung cancers^12^,^18^. In this study, we determined the binding sites of KRT19 and HER2 and investigated the impact of KRT19 and HER2 interactions in signal transduction pathways to decode their possible roles in oncogenesis.

## Results

### Detection of KRT19 as a HER2-binding protein

To determine novel HER2-binding protein candidates in lung cancers, we used an immunoprecipitation and mass spectrometry analysis. Several lung cancer cell lines and human embryonic kidney cells (HEK293T) were transfected with HA-tagged wild type or TD mutant *HER2*. Each cell lysate sample was immunoprecipitated with anti-HA tag beads, and the resulting samples were electrophoresed. Although many proteins appeared in a similar fashion with various combination of cell lines or transfectants, one clear band (40 kDa) was identified when immunoprecipitated HEK293T were compared with A549 (Supplementary Fig. S1). This band was analyzed using mass spectrometry and was shown to be KRT19.

**Figure 1:**
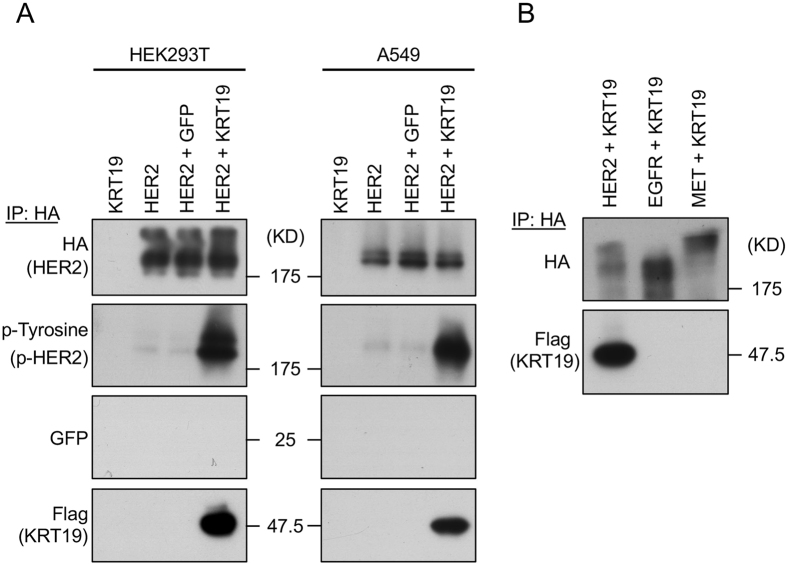
The interaction of KRT19 and HER2. (**A**) KRT19 and/or HER2 were transfected to HEK293T and A549. Co-transfection of HER2 and KRT19 was necessary to phosphorylate HER2. (**B**) HEK293T cells were co-transfected with KRT19 and one of the receptor tyrosine kinases (HER2, EGFR or MET) to investigate the specificity of the receptor protein that bound to KRT19. Each protein sample was immunoprecipitated with anti-HA-tag beads. KRT19 only bound to HER2. Induced HER2, EGFR and MET were tagged with HA, and KRT19 was tagged with Flag. Full-length blots are presented in Supplementary Fig. S9.

To investigate the ability of KRT19 to bind HER2, we co-transfected *HER2* and *KRT19* into HEK293T and A549 cells, respectively. Protein samples were immunoprecipitated using anti-HA tag beads. The results of Western blotting showed that the binding of KRT19 to HER2 contributed to HER2 phosphorylation in serum free condition (Fig. 1A). Although artificially expressed, HER2 alone was not phosphorylated, while the HER2 that had bound to KRT19 was phosphorylated in both the HEK293T and A549 cells (Fig. 1A). We co-transfected *KRT19* with several kinds of oncogenic receptors (*HER2, EGFR, and MET*) into HEK293T cells to examine the specificity of KRT19 binding (Fig. 1B). Among the receptor tyrosine kinases that were tested, KRT19 only bound to HER2.

### HER2 and KRT19 expressions in lung cancer cells

**Figure 2:**
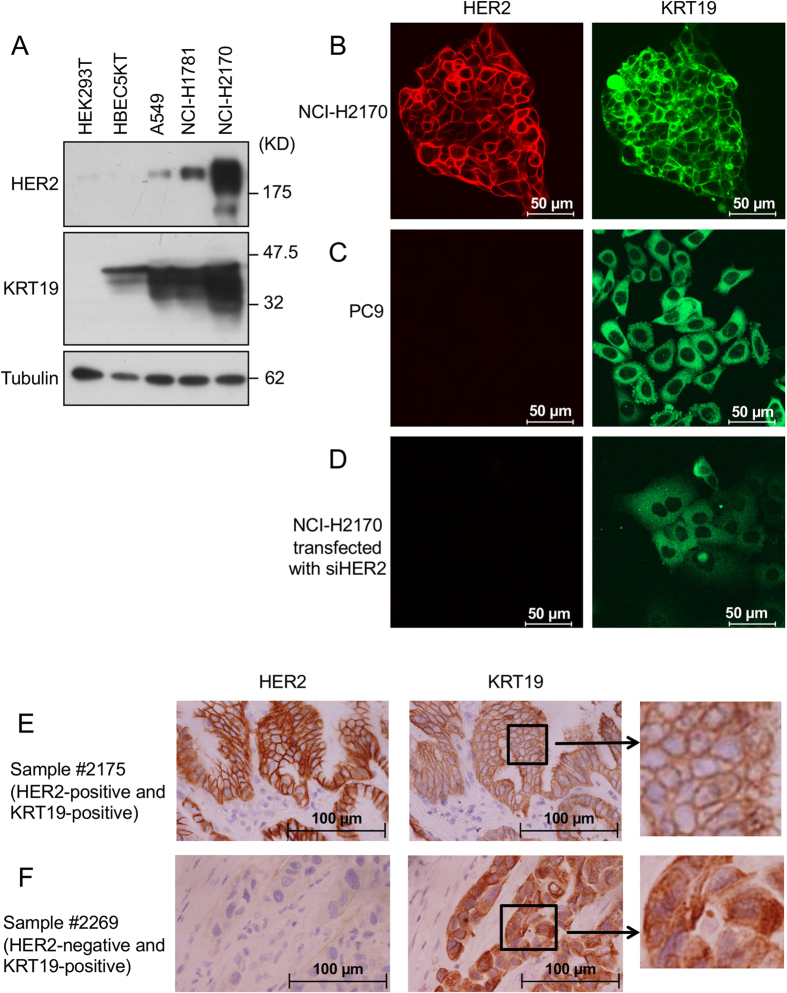
Immunofluorescent and immunohistochemical staining in lung cancer cell lines and primary tumors. (**A**) Expression levels of HER2 and KRT19 in HEK293T, HBEC5KT, A549 (*HER2*-wild type), NCI-H1781 (*HER2*-mutant), and NCI-H2170 (*HER2*-amplified) are shown. (**B**) NCI-H2170 cells. (**C**) PC9 cells. (**D**) HER2 knock downed NCI-H2170 with siRNA. (**E**) HER2-positive and KRT19-positive primary lung cancer. (**E**) HER2-negative and KRT19-positive primary lung cancer. Location of KRT19 was different according to the expression of HER2 in both cell lines and clinical samples. Full-length blots are presented in Supplementary Fig. S9.

We examined the HER2 and KRT19 expression levels in the HEK293T, human bronchial epithelial cell line (HBEC5KT), A549, NCI-H1781, and NCI-H2170 cell lines. KRT19 was highly expressed in the *HER2*-mutant NCI-H1781 cell line (G776V, Cins^6^) and the *HER2*-amplified NCI-H2170 cell line (copy number: 135^19^), when compared with its expressions in the HEK293T or HBEC5KT cell lines (Fig. 2A). Next, we examined KRT19 localization in lung cancer cell lines and primary tumors by using immunofluorescent staining. KRT19 was localized to the cell membrane in NCI-H2170 cells that highly expressed both HER2 and KRT19 (Fig. 2B). Conversely, in PC-9 cells with a negative expression of HER2, KRT19 was localized mainly in the cytoplasm (Fig. 2C). Interestingly, when we used siRNA to knock down HER2 expression in NCI-H2170 cells, KRT19 diffused to the cytoplasm from the cell membranes (Fig. 2D).

To obtain a clue toward solving the mechanism by which KRT19 is able to translocate to the HER2-positive cell membrane in lung cancers, we attempted to examine a phosphorylation status of the KRT19 protein. We used the avidin-beads with the biotin-conjugated KRT19 antibody to the immunoprecipitation of the intrinsic KRT19 protein in the cell lysate. This immunoprecipitation method is useful to collect antibody free sample after elution procedure with acidic buffer because of significantly strong interaction between avidin and biotin components. Using this, we found that KRT19 was appeared as single band in both HER2 negative PC-9 and HER2 positive NCI-H2170 cells at almost similar level and the protein was not detected in the normal human OUMS-24 fibroblasts. When we compared these bands in S/T-phosphorylation, we figured out the phosphorylation state was markedly increased in the KRT19 in NCI-H2170 cells compared to that in PC-9 cells, suggesting that phosphorylation modification might play a role in membrane transport of the KRT19 in HER2-overexpressing lung cancers (Supplementary Fig. S2).

To investigate the *in vivo* distribution of KRT19 observed in the artificial *in vitro* system, we used immunohistochemical staining to examine the association between KRT19 expression and the localization and HER2 expression status in the surgically resected primary lung cancer tissues. Among 86 cases, KRT19-positive expression was found in 70 cases (47 cases of Score 2+ and 23 cases of Score 3+). HER2 positive expression was found in 37 cases (33 cases of Score 2+ and 4 cases of Score 3+). HER2 was significantly expressed in KRT19-positive tumors (36/70, 51.4%) compared with KRT19-negative tumors (1/16, 6.3%) (*P* < 0.001) (Supplementary Table S1). Furthermore, we focused on the location of KRT19 in KRT19-positive tumors. Among 70 KRT19-positive tumors, KRT19 expression was found at the cell membrane in 57 tumors and at the cytoplasm in 13 tumors, respectively. KRT19 was located at the cell membrane significantly more frequently in HER2-positive rather than in HER2-negative tumors (KRT19-positivity in cell membrane, HER2-positive vs. HER2-negative; 34/36 [94.4%] vs. 23/34 [67.6%], *P* = 0.0052) (Supplementary Table S1 and Fig. 2E and F). Of note, no significant associations were observed between *EGFR* mutation and HER2 expression or the KRT19 expression status (data not shown). These results suggest that HER2 affects the localization of KRT19, and HER2 and KRT19 co-expression may have an important role in HER activation in lung cancer cells.

To strengthen the result of different localization patterns we observed in the immunocytochemistry pictures, we then conducted cell fractionation of cells into membrane and cytosol enriched fractions. By this approach, we found that KRT19 was present mainly in cytosol in HER2-negative PC-9 cells. However, the protein was detected in HER2-positive membrane fraction with higher level in NCI-H2170 cells. These results indicate that membrane localization of KRT19 is dependent on the strong appearance of HER2 at cellular membrane (Supplementary Fig. S3).

### Determination of the binding domains between KRT19 and HER2

**Figure 3:**
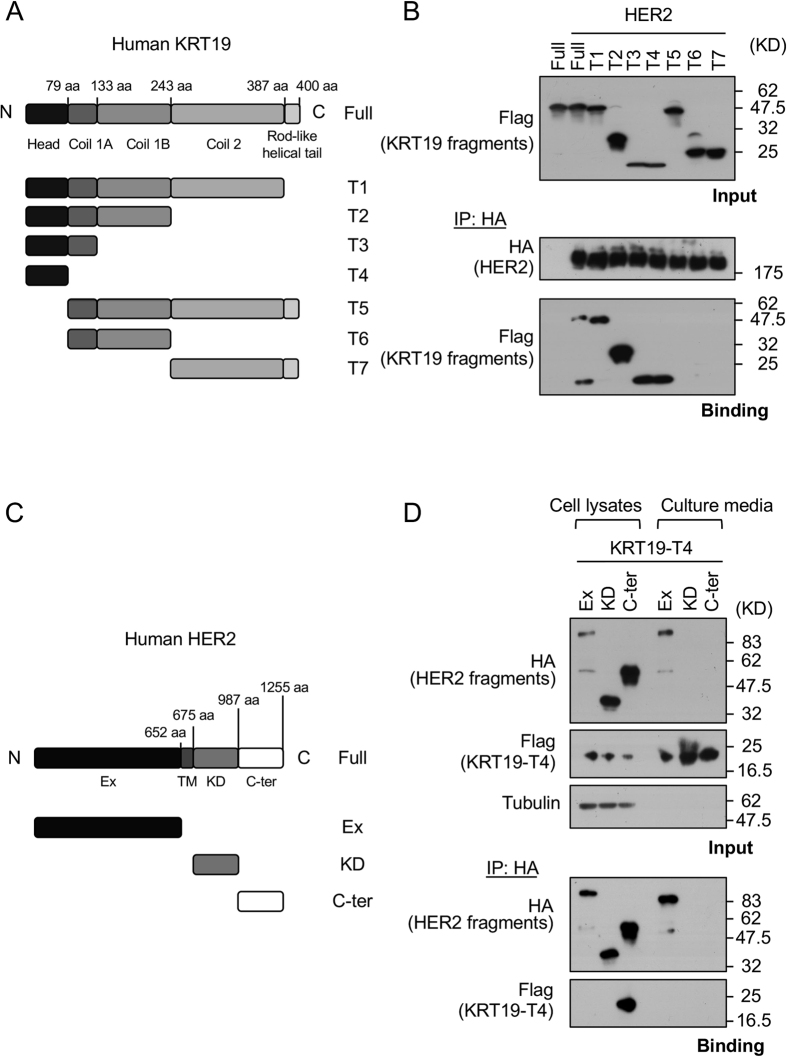
The binding domains of KRT19 and HER2 identified by the co-transfection of KRT19 and HER2. (**A**) The truncated variants of KRT19 were constructed. The detail is as follows: Full, full length of KRT19; T1, head, coil 1 A, coil 1B and coil 2; T2, head, coil 1 A and coil 1B; T3, head and coil 1 A; T4, head; T5, coil 1 A, coil 1B, coil 2 and rod-like helical tail; T6, coil 1 A and coil 1B; and T7, coil 2 and rod-like helical tail. (**B**) HER2 and each variant of KRT19 were transfected to HEK293T. HER2 complexes were collected using anti-HA tag immunoprecipitation. Only T4-including variants of KRT19 were able to bind to HER2. (**C**) The truncated variants of HER2 were constructed. The detail is as follows: Full, full length of HER2; Ex, extracellular domain; KD, kinase domain; and C-ter, COOH-terminal domain. (**D**) KRT19-T4 and each truncated variant of HER2 were co-transfected into HEK293T. These transfected cell lysates and their culture media were immunoprecipitated with anti-HA-tag beads. KRT19 only binds to intracellular HER2. Full length and fragments of HER2 were tagged with HA, and full length and fragments of KRT19 were tagged with Flag. Full-length blots are presented in Supplementary Fig. S9.

Next, we focused on the binding domain of KRT19 involved in binding to HER2. *KRT19* consists of five domains: head, coil 1 A, coil 1B, coil 2 and rod-like helical tail domains (http://www.uniprot.org/uniprot/P08727). According to the domain composition, we constructed seven kinds of truncated variants by a combining of these five domains and naming them T1 to T7 (Fig. 3A). We then co-transfected *HER2* and each *KRT19* variant into HEK293T cells and performed binding assays. Although the truncated T1‒T4 variants of KRT19 bound to HER2 as well as to the full length of KRT19, the T5–T7 variants of KRT19 did not bind to HER2 (Fig. 3B). These results showed that the NH2-terminal head domain of KRT19 acted as a binding domain for HER2. Regarding binding site of HER2, we designed three kinds of HA-tagged HER2 truncated variants: 1) extracellular domain (Ex), 2) kinase domain (KD), and 3) COOH-terminal domain (C-ter) (Fig. 3C). We co-transfected each *HER2* truncated variant and the truncated variant of KRT19-T4 (the binding domain of KRT19) into HEK293T cells in serum free condition. Flag-tagged KRT19-T4 was detected in the immunoprecipitated C-ter protein sample of HER2 (Fig. 3D) in cell lysates not in culture media.

To examine the direct and specific interaction between HER2 C-terminal domain and KRT19 at N-terminal T4 domain, we prepared a series of purified recombinant proteins, GST, GST-KRT19 (full length), GST-T4 and Flag-tagged HER2 C-terminal domain and analyzed the binding of GST fusion proteins to the HER2 C-terminal domain. As shown in Supplementary Fig. S4, HER2 C-terminal domain showed direct binding to both GST-KRT19 (full length) and GST-T4, but not GST alone.

**Figure 4:**
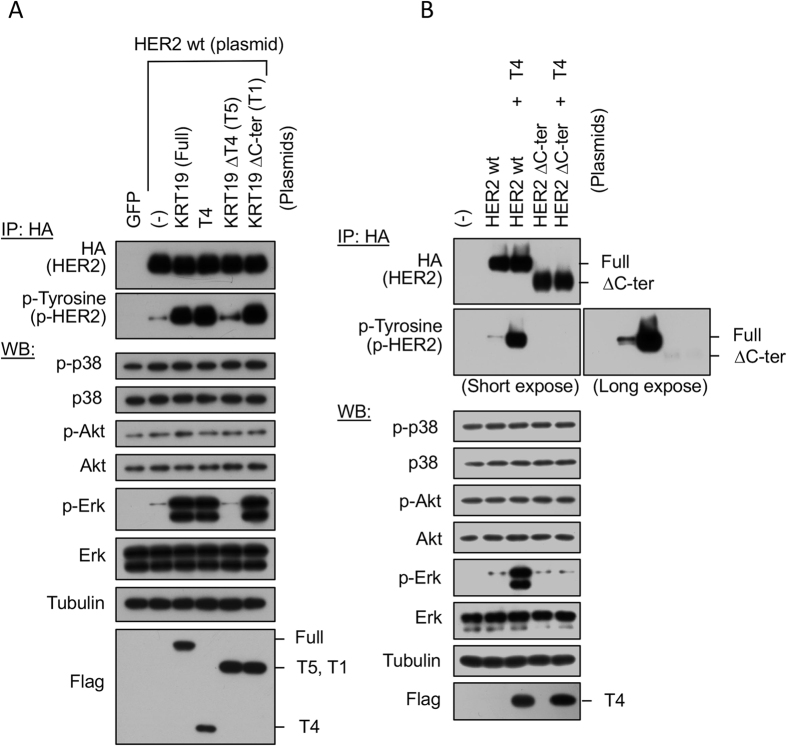
T4 domain in KRT19 has an important role for HER2 activation. (**A**) Activation profiles for HER2 and downstream effector kinases. HEK293T cells were transfected with various couples of the indicated plasmids, lysed and subjected to immunoprecipitation using the anti-HA agarose beads and following Western blotting with either anti-HA antibody or with anti-phospho-Tyrosine antibody. Using the whole cell extracts as described above without immunoprecipitation, Western blot was carried out to assess the phosphorylation levels of the indicated effector kinases (p38, Akt and Erk). (**B**) HEK293T cells were also transfected with various couples of the indicated plasmids, lysed and subjected to immunoprecipitation using the anti-HA agarose beads and following Western blotting with either anti-HA antibody or with anti-phospho-Tyrosine antibody. Western blot was carried out to assess the phosphorylation levels of the indicated effector kinases (p38, Akt and Erk) using the whole cell extracts beforehand the immunoprecipitation. Full-length blots are presented in Supplementary Fig. S10.

To avoid the issue that short fragment of protein often shows unexpected artificial function, we further attempted to examine the activation levels of effector kinases controlled by HER2 after transfection with T4-deleted KRT19 (T5) and C-terminal deleted KRT19 (T1). Using these deleted forms of KRT19, we found that T5 but not T1 significantly reduced the Erk phosphorylation in parallel with down-regulation of HER2 phosphorylation. Furthermore, T4 fragment was never able to induce the phosphorylation of HER2 and its concomitant activation of Erk when HER2 C-terminal site, T4-interacting region, was deleted (HER2 ΔC-ter). Taken together, we strongly indicate that T4 domain in KRT19 has a significant role for HER2 activation through the binding with HER2 C-terminal domain in intracellular space (Fig. 4).

These results indicated that the HER2-C-ter was a binding domain for KRT19, and the binding of HER2 to KRT19 occurred in the cytoplasm. For this point, Ju *et al*. showed that KRT19 was released into the extracellular environment and binds to the extracellular domain of HER2^20^. They also reported that extracellular phosphorylated form of KRT19 at Serine 35 was important for HER2 activation^20^, thus we tried to stimulate HER2-overexpressed HEK293T cells with GST-KRT19 (wt and S35D: Serine 35 was replaced to Aspartic acid) and GST-T4 (wt and S35D). The mutant S35D stands for a representative mimic of phosphorylated form. When we treated the cells with the purified proteins, we found that full-length of KRT19 (wt) induced HER2 phosphorylation at significant level and further activation of HERs was observed by the treatment with mutant type S35D. Interestingly, these phenomena were not observed in the cells with either T4 wt- or mut S35D treatment. Collectively, these results indicate that KRT19 acts to HER2 not only intracellularly but also extracellularly and that T4 domain is required for intracellular function of KRT19 to activate HER2 (Supplementary Fig. S5).

### Downstream signal activation of HER2 with KRT19 or ligands

**Figure 5:**
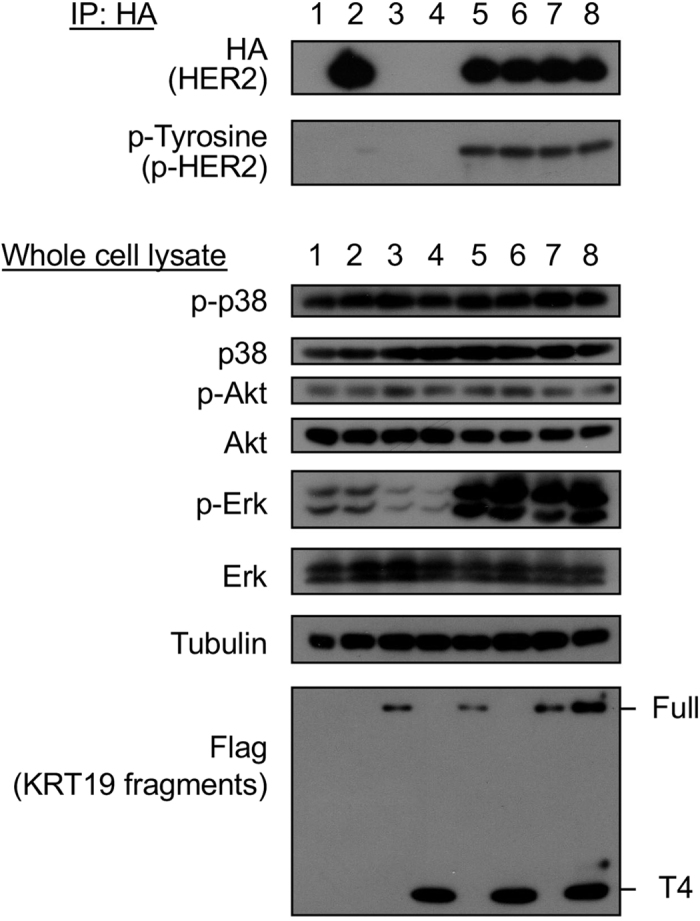
Co-transfection of HER2, full length of KRT19 and/or T4 variants to HEK293T cells. Lanes 1–8 represents respective proteins tranfected to HEK293T as follows: lane 1, GFP; lane 2, HER2; lane 3, KRT19 (full length); lane 4, T4 (head domain of KRT19); lane 5, HER2 and KRT19; lane 6, HER2 and T4; lane 7, HER2, KRT19 and GFP; lane 8, HER2, KRT19 and T4. The samples for HA and phospho-Tyrosine were collected using anti-HA tag immunoprecipitation. Transfection of KRT19 enhanced the expression of phospho-HER2 as well as phospho-Erk in HER2-transfected cells. T4, head domain of KRT19, was enough to activate HER2 and Erk. Full-length blots are presented in Supplementary Fig. S9.

To investigate the effects on downstream pathway molecules caused by KRT19 and HER2 binding, we co-transfected *HER2*, the full length of *KRT19*, and/or *KRT19*-T4 into HEK293T cells and phosphorylation of p38, Akt, and Erk were examined. The combination of HER2 and KRT19-T4 as well as the combination of HER2 and the full length of KRT19 phosphorylated HER2 and Erk (Fig. 5).

**Figure 6:**
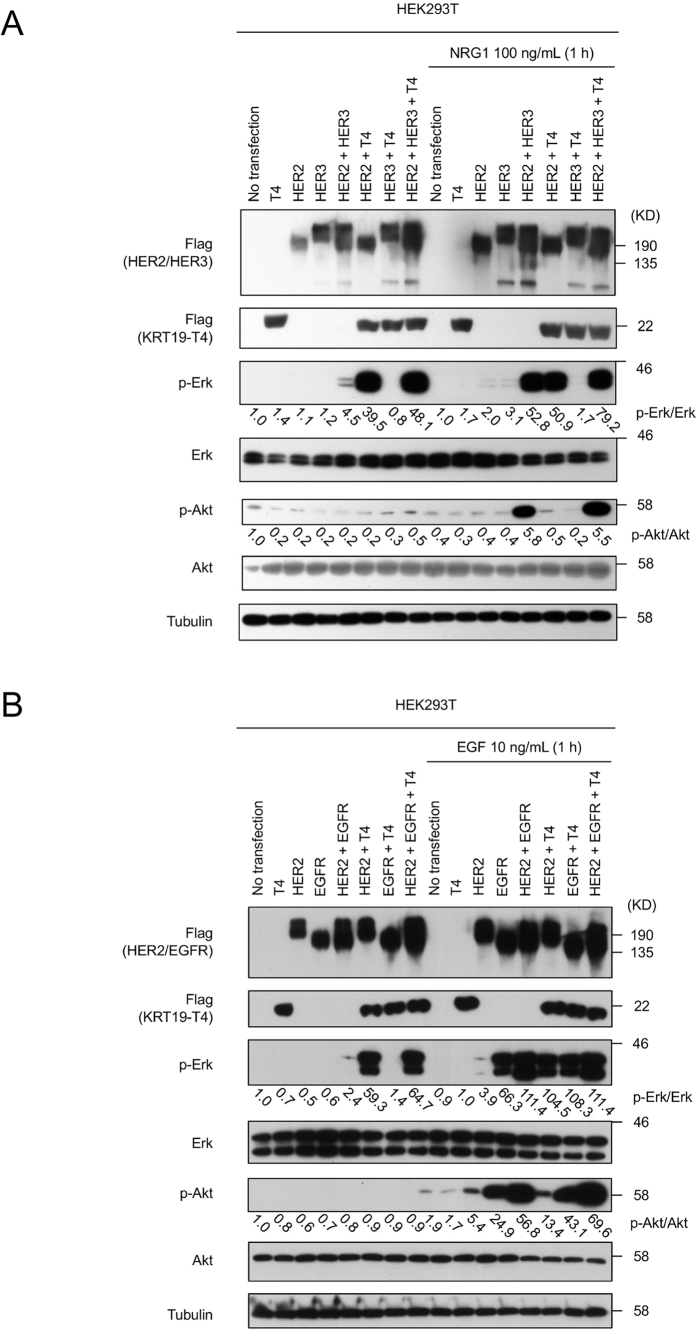
Downstream signal activation of HER2 with ligands. (**A**) Erk was activated through the binding of HER2 to KRT19-T4 whereas Akt is activated through the heterodimerization of HER2 with HER3. KRT19-T4, HER2 and HER3 were independently or simultaneously transfected to HEK293T and the cells were kept for 24 h under serum free condition before treated or not treated with NRG1, a representative ligand for HER3. Expression of p-Erk was strongly increased through the co-transfection of HER2 and KRT19-T4, or the co-transfection of HER2 and HER3 with the stimulation of NRG1 (100 ng/ml for 1 h). On the other hand, expression of p-Akt was strongly increased through the co-transfection of HER2 and HER3 with the stimulation of NRG1. KRT19-T4 has a pivotal role to activate Erk through its binding to HER2. Induced HER2, HER3 and KRT19-T4 were tagged with Flag. Each level of phospho-Erk and phospho-Akt was expressed as p-Erk/Erk and p-Akt/Akt ratio, respectively, after normalization with Tubulin. (**B**) Erk was activated through the binding of HER2 to KRT19-T4 whereas Akt is activated through the heterodimerization of HER2 with EGFR. KRT19-T4, HER2 and EGFR were independently or simultaneously transfected to HEK293T and the cells were kept for 24 h under serum free condition before treated or not treated with EGF, a representative ligand for EGFR. Expression of p-Erk was strongly increased through the co-transfection of HER2 and KRT19-T4, or the co-transfection of HER2 and EGFR with the stimulation of EGF (10 ng/ml for 1 h), the ligand for EGFR. EGFR alone with EGF increased p-Erk modestly. On the other hand, expression of p-Akt was strongly increased through the co-transfection of HER2 and EGFR with the stimulation of EGF. EGFR alone with EGF increased p-Akt modestly. KRT19-T4 has a pivotal role to activate Erk through its binding to HER2. Induced HER2, EGFR and KRT19-T4 were tagged with Flag. Each level of phospho-Erk and phospho-Akt was expressed as p-Erk/Erk and p-Akt/Akt ratio, respectively, after normalization with Tubulin. Full-length blots are presented in Supplementary Fig. S9.

Next, we investigated the relationship among HER2, HER3 and KRT19, since HER3 is considered to be the preferred partner of HER2, and HER2-HER3 dimers are thus known to be the most active of the possible HER family dimers^21^. We transfected *HER2, HER3*, and *KRT19*-T4 independently or simultaneously into HEK293T cells (Fig. 6A). The combination of *HER2* and *KRT19*-T4 activated Erk irrespective of *HER3* without the stimulation of heregulin beta -1 (NRG1), the ligand for HER3, whereas the combination of *HER2* and *HER3* did not activate Erk. Under the stimulation with NRG1, the combination of *HER2* and *HER3* activated both Erk and Akt; these findings are concordant with a previous report describing the activation of the PI3K-Akt pathway through HER3^22^.

Then, we also investigated the relationship among *EGFR, HER2*, and *KRT19*, since the interaction between EGFR and HER2 plays an important oncogenic role in NSCLC^23^,^24^. We transfected *HER2, EGFR*, and *KRT19*-T4 independently or simultaneously into HEK293T cells (Fig. 6B). Erk was, again, strongly activated through the co-transfection of *HER2* and *KRT19*-T4 as well as the co-transfection of *HER2* and *EGFR* with epidermal growth factor (EGF) (ligand for EGFR) stimulation. The transfection of *EGFR* alone with EGF increased p-Erk modestly. On the other hand, Akt was strongly activated through the co-transfection of *HER2* and *EGFR* with EGF stimulation. The transfection of *EGFR* alone with EGF increased p-Akt modestly. The ratio between p-Erk and Erk, and the ratio between p-Akt and Akt were calculated after the normalization with Tubulin. We put the calculated value of them below the band in Fig. 6.

### HER2 signaling and cell growth under KRT19 suppression

**Figure 7:**
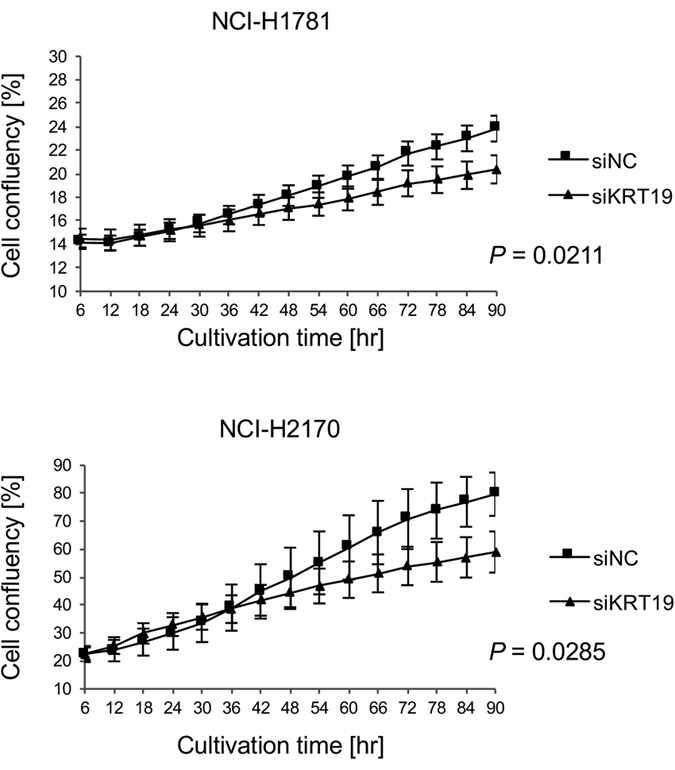
HER2 signaling and cell growth under KRT19 suppressed conditions. The KRT19 in NCI-H1781 and NCI-H2170 were suppressed using siRNA. Cell growth was assessed using IncuCyte ZOOM apparatus. Cell growth was inhibited by the knockdown of KRT19.

To investigate the role of KRT19 on the endogenous HER2 phosphorylation status and the subsequent Erk signaling, we suppressed KRT19 using siRNA in the NCI-H1781 and NCI-H2170 cell lines (Supplementary Fig. S6). Under KRT19-suppressed conditions, the phosphorylation of both HER2 and Erk was downregulated in the NCI-H1781 and NCI-H2170 cell lines, and the total amount of HER2 protein slightly decreased (density ratio of HER2 to p-HER2 [Tyr 1221/1222] was 0.572 in NCI-H2170 cell lines and 0.780 in the NCI-H1781 cell lines, while that of HER2 to p-HER2 [Y877] was 0.735 in the NCI-H2170 cell lines and 0.672 in the NCI-H1781 cell lines.). Furthermore, we investigated the cell proliferation activities of these cell lines (Fig. 7). We evaluated the cell proliferation activity as the cell confluency of each well. At 90 h after siRNA transfection for KRT19, the cell proliferation activity had decreased in both the NCI-H1781 (*P* = 0.0211) and the NCI-H2170 (*P* = 0.0285) cell lines compared with the control siRNA.

### Prognostic significance of KRT19 mRNA expression

We further investigated whether the overexpression of KRT19 has any impact on the clinicopathological characteristics of lung cancer. To this end, using two independent datasets, including 226 stage I–II adenocarcinomas of the National Cancer Center in Japan (NCC) and 230 stage I–IV adenocarcinomas of the cancer genome atlas (TCGA), we examined the relationship between *KRT19* mRNA expression and the outcomes of lung adenocarcinoma patients. The patients were divided into four groups according to the low and high expressions of both *HER2* mRNA and *KRT19* mRNA. In NCC patients with a higher expression of *HER2*, the overall survival (OS) in cases with a higher expression of *KRT19* was significantly shorter than that in *KRT19* cases with a lower expression (*P* = 0.0139) (Supplementary Fig. S7A). In addition, the same trend was observed in the TCGA dataset, although the statistical value was marginal (*P* = 0.0613) (Supplementary Fig. S7B), suggesting that the cooperative expression of *KRT19* and *HER2* might be correlated with a poor prognosis in lung adenocarcinoma patients.

### Gene set enrichment analysis

We performed a Gene Set Enrichment Analysis (GSEA) using the NCC and TCGA datasets for NSCLC. Among cases in which the molecular signatures database of hallmark gene sets was used, “HALLMARK_GLYCOLYSIS” was statistically significantly enriched as the number one gene set in both the NCC (familywise error rate [FWER], *P* value < 0.01; Supplementary Fig. S8A) and the TCGA (FWER, *P* value = 0.05; Supplementary Fig. S8B) datasets. Furthermore, among cases in which the molecular signatures database of C2 curated gene sets was used, the Ras-related gene set was significantly enriched in both the NCC (FWER, *P* value = 0.04; Supplementary Fig. S8C) and the TCGA (FWER, *P* value = 0.01; Supplementary Fig. S8D) datasets with the top rank in total score (Supplementary Table S2). Notably, these Ras-related genes have been reported to contribute to carbon metabolism^25^. Further studies are warranted to determine how the cooperative expressions of KRT19 and HER2 are associated with the induction of carbon metabolism.

## Discussion

In the current study, we found that KRT19 bound to HER2 intracellularly followed by the subsequent activation of HER2 and its downstream Erk protein, indicating the role of KRT19 as an oncogenic molecule. Furthermore, we found that the expression of HER2 was correlated with the expression of KRT19, and the location of KRT19 was affected by the existence of HER2 in lung cancer cells, supporting the intracellular binding of KRT19 and HER2. The poor prognosis of NSCLC cases with HER2 and KRT19 overexpression as identified using publicly available databases further strengthened our findings of the cooperative effect of HER2 and KRT19 on malignant behavior in double-positive lung cancers.

The mechanism by which KRT19 is translocated to the plasma membrane of HER is still in enigma in detail. In this issue, we are considering that KRT19 automatically enriches at inside surface of plasma membrane when HER2 is overexpressed since T4 is able to bind directly to HER2 cytoplasmic tail irrespective to phosphorylation state at Serine35. On the other hand, we had another interesting phenomenon that the S/T-phosphorylation state of KRT19 was upregulated in HER2-overexpressed NCI-H2170 cells. This suggests that phosphorylation of KRT19 may also contribute translocation mechanism of KRT19 to HER2 cytoplasmic tail at plasma membrane. Further studies would be required to include or exclude the physiological importance of the phosphorylation in delivery of KRT19 to plasma membrane.

The role of keratins in lung cancers has not been well elucidated. The keratins are intermediate filaments subdivided into cytokeratins and hair keratins. They are classified into two types: low-molecular weight acidic type I keratins and high-molecular weight basic or neutral type II keratins. The major cellular functions of cytokeratins are mechanical support, cytoarchitecture, cell migration, and signal modulation^26^. Among keratins, the KRT19 is the smallest member of the type I keratin family and is known as a tumor marker in several kinds of malignancies^12^,^13^,^14^,^15^,^16^,^17^,^18^. Regarding the function of KRT19 in cancers, the expression of KRT19 might contribute to the invasiveness of hepatocellular carcinoma (HCC)^27^. Another study showed that KRT19-positive HCC demonstrated self-renewal ability and acted as a stem cell marker^28^. Regarding the binding of KRT19 to HER2, our results indicated that HER2-C-ter, the tail domain, was the binding domain for KRT19 and that the binding occurred in the cytoplasm. Indeed, the fact that KRT19 is localized to the cell membranes in HER2-positive lung cancer cells supports the hypothesis that KRT19 binds to HER2 and activates it at the intracellular level, resulting in the further activation of downstream effectors. Of note, similar interactions between some cytokeratins and transmembrane receptors have been demonstrated. For example, cytokeratin 8 and cytokeratin 18 both bind to cytoplasmic tail domain of the tumor necrosis factor receptor 2 to moderate TNF-induced Jun NH2-terminal kinase intracellular signaling and NFkB activation^29^. Of note, binding site of cytokeratin 18 is in its NH2-terminal half^29^.

While the ligand of HER2 has not been identified, the specific ligands for EGFR and HER3 are known. In a downstream signal study of HER family members with KRT19-T4 or their ligands, the binding of HER2 to KRT19 led to activation of Erk not Akt. These results are consistent with the well-known fact that the HER2 activates Erk pathways, but not Akt pathways^30^. On the other hand, Akt, as well as Erk, was activated through the co-transfection of HER2 and HER3 with NRG1 or HER2 and EGFR with EGF, indicating that each ligand bound to their target receptors followed by their downstream activation. In addition, our results also suggested that the binding of KRT19-T4 to HER2 did not contribute to the heterodimerization of HER2 with EGFR or HER3, since only Erk, and not Akt, was activated, possibly in response to the interaction of HER2 and KRT19-T4 in our experimental system.

Our studies confirmed the results by Ju *et al*.^20^, that extracellular KRT19 especially its phosphorylated form at Serine35 played a significant role on HER2 activation through an extracellular binding between them. In addition to this, we discovered that the interaction of KRT19 and HER2 was not restricted to extracellular space, i.e., the interaction was also happened intracellularly. The intracellular coupling of them was formed via T4 side of KRT19 and C-terminal side of HER2, eventually leading to HER2 activation with much higher level. We hence support the idea of these interactions of KRT19 to HER2 at both inside and outside of cells.

As KRT19 binds to HER2 intracellularly and leads to the subsequent phosphorylation of HER2 and Erk, targeting KRT19 is a potential therapeutic option for *HER2*-driven cancers. This fact suggests that inhibitors of KRT19-T4 binding could be used as therapeutic compounds, resulting in new therapeutic strategies for cancer treatment.

As mentioned, using the publicly available mRNA expression profile database, we found that the cooperative expression of *KRT19* and *HER2* may be associated with a poor prognosis in lung cancer patients. Besides bench works, further bioinformatics analysis may also shed light on the association between the cooperative expression of KRT19 and HER2 and the induction of carbon metabolism. Also, some ion channel genes were included among the Ras-related genes, such as the KCa 3.1 channel gene (*KCNN4*). This gene was highly expressed in patients with the cooperative induction of KRT19 and HER2 (data not shown). Of interest, KCNN4 has been reported to be a marker of poor prognosis in NSCLC patients^31^. Further studies should be performed to understand how the cooperative expression of KRT19 and HER2 contributes to malignant potential of lung cancer.

In conclusion, we found that the NH2-terminal head domain of KRT19 bound to the COOH-terminal domain of HER2 in HER2-activated lung cancer; this process induces HER2 phosphorylation and the subsequent activation of Erk. These findings suggest that KRT19 could be a therapeutic target for lung cancer. Further studies may lead to establish a new therapeutic strategy targeting KRT19 and HER2 in HER2-activated malignant tumors including lung cancer.

## Materials and Methods

### Cell lines and reagents

HEK293T, HBEC5KT, OUMS-24, and human lung cancer cell lines A549, NCI-H1781 (*HER2*-mutant), NCI-H2170 (*HER2*-amplified), and PC-9 were used in this study. HBEC5KT, NCI-H1781, and NCI-H2170 were provided by Dr. Adi F. Gazdar (Hamon Center for Therapeutic Oncology Research and Department of Pathology, University of Texas Southwestern Medical Center at Dallas, Dallas, TX), who had previously established these cell lines^19^,^32^. A549 was purchased from American Type Culture Collection (Manassas, VA). PC-9 cells were obtained from Immuno-Biological Laboratories (Takasaki, Gunma, Japan). HEK293T cells were obtained from RIKEN Bio Resource Center (Tsukuba, Ibaraki, Japan). OUMS-24 is a normal human fibroblast cell line, which was provided by Dr. Masayoshi Namba (Emeritus Professor of Cell Biology, Okayama University Graduate School of Medicine, Dentistry and Pharmaceutical Sciences, Okayama, Japan), who had previously established this cell line^33^. These cell lines were proven to have individual genetic origins using the Powerplex 1.2 system (Promega, Madison, WI) at the University of Texas Southwestern Medical Center at Dallas. HEK293T cells were maintained in Dulbecco’s modified Eagle’s medium (DMEM) supplemented with 10% heat-inactivated fetal bovine serum. A549, NCI-H2170, NCI-H1781, and PC-9 cells were maintained in RPMI-1640 medium supplemented with 10% heat-inactivated fetal bovine serum. HBEC5KT cells were maintained in Keratinocyte-SFM medium with bovine pituitary extract and human recombinant EGF. All cells were cultured at 37.0 °C with 5% CO_2_. All cell lines were tested for mycoplasma contamination by using Venor^®^GeM OneStep (Minerva Biolabs, Berlin).

### Western blot analysis, Immunoprecipitation, and ligand stimulation

The detailed protocol for Western blotting has been described previously^34^. In brief, whole cell extracts were prepared by using a commercialized lysis reagent, M-PER (78501. Thermo Fisher Scientific, Waltham, MA). Enrichment and following extraction of proteins of cytoplasm and membrane fractions in cultured cells were also prepared by using a cell fractionation kit (#9038. Cell Signaling Technology, Beverly, MA) according to the manufactures instruction. The primary antibodies were as follows: anti-HA (Human influenza hemagglutinin) mouse antibody (#2367. Cell Signaling Technology), anti-phospho-Tyrosine HRP (HAM1676. R&D Systems, Minneapolis, MN), anti-GFP mouse antibody (#2955. Cell Signaling Technology), anti-Flag mouse antibody (F1804. Sigma-Aldrich, St. Louis, MO), anti-Cytokeratin 19 mouse antibody (sc-6278. Santa Cruz Biotechnology, Dallas, TX), anti-Tubulin mouse antibody (T5138. Sigma-Aldrich), anti-HER2/ErbB2 rabbit antibody (#4290. Cell Signaling Technology), anti-phospho-HER2/ErbB2 (Tyr1221/1222) rabbit antibody (#2243. Cell Signaling Technology), anti-phospho-HER2/ErbB2 (Y877) rabbit antibody (#2241. Cell Signaling Technology), anti-p44/p42 MAPK (mitogen-activated protein kinase) (Erk) rabbit antibody (#9102. Cell Signaling Technology), anti-phospho-p44/p42 MAPK (phospho-Erk) rabbit antibody (#4370. Cell Signaling Technology), anti-Akt rabbit antibody (#9272. Cell Signaling Technology), anti-phospho-Akt (Ser473) rabbit antibody (#9271. Cell Signaling Technology), anti-p38 rabbit antibody (#8690. Cell Signaling Technology), anti-SOD1 mouse antibody (M062-3. MBL, Nagoya, Japan), anti-MET rabbit antibody (sc-10, Santa Cruz Biotechnology), and anti-phospho-p38 rabbit antibody (#4511. Cell Signaling Technology). The following secondary antibody was used: goat anti-mouse (sc-2031) or anti-rabbit (sc-2030) immunoglobulin G conjugated with horseradish peroxidase (Santa Cruz Biotechnology). To detect specific signals, the membranes were examined by using ECL plus Western blotting detection reagents (GE Healthcare, Milwaukee, WI). Monoclonal Anti-HA tag-agarose (A2095. Sigma-Aldrich) and Glutathione sepharose 4B (GSH beads, 17075601. GE Healthcare) were used for the immunoprecipitation experiments. Anti-Cytokeratin 19 mouse antibody (sc-6278. Santa Cruz Biotechnology) was biotinylated using a Biotin Labeling Kit-SH (LK03. Dojindo Molecular Technologies, Rockville, MD) to recover antibody-free samples after immunoprecipitation using streptavidin-agarose (SA10004. Thermo Fisher Scientific). EGF (Sigma-Aldrich) and NRG1 (Sigma-Aldrich) were used as the ligands for EGFR and HER3, respectively.

### Plasmid vectors

pIDT-SMART (C-TSC) plasmid vector, named pCMViR-TSC^35^, was used for forced gene expression. Inserted human cDNAs are listed below. Human cDNAs encoding full-length wild type *HER2* and its truncated variants (Ex: 1–652 aa, KD: 720–987 aa, C-ter: 988–1255 aa, ΔC-ter: 1–987 aa), TD mutant *HER2*, full-length *EGFR* and *MET* were designed for expression as COOH-terminal HA-6His-tagged forms. Full-length *HER2*, EGFR and *HER3*, were also prepared to express as COOH-terminal Flag-6His-tagged forms. Human cDNA encoding full-length KRT19 and its truncated variants (T1: 1–387 aa, T2: 1–243 aa, T3: 1–133 aa, T4: 1–79 aa, T5: 80–400 aa, T6: 80–243 aa, T7: 244–400 aa) were designed for expression as COOH-terminal Flag-6His-tagged forms. Transient transfection of the prepared plasmids into cultured cells was performed using FuGENE-HD (Promega BioSciences, San Luis Obispo, CA).

### Purification of recombinant KRT19-T4 (T4) and HER2 C-ter (C-ter) fragments from mammalian expression system

The T4 and C-ter expression constructs (pCMViR-TSC-T4-3xFlag-6His and pCMViR-TSC-C-ter-3xHA-6His) was transiently transfected to HEK293T cells by FuGENE-HD transfection reagent (Promega BioSciences) under serum-free conditions using Opti-MEM medium (Life Technologies, Carlsbad, CA). Forty-eight hours after the transfection, the cells were collected, lysed, and subjected to affinity chromatography with a monoclonal anti- DYKDDDDK antibody bead column for T4 expression and with a monoclonal anti- HA antibody bead column for C-ter expression, which capture Flag-tagged proteins (012-22781. WAKO, Tokyo, Japan) and HA-tagged proteins (A-2095. Sigma-Aldrich), respectively.

### Purification of recombinant GST-KRT19 and GST-T4 fusion proteins from bacterial expression system

Full-length of human KRT19 cDNAs (wt and mut S35D, which displays mimic of phosphorylated form) and T4 fragment cDNAs (wt and mut S35D) were cloned into the pGEX-6P1 vector (GE Healthcare) for expression in *E. coli* as the GST fusion forms. Purification of the bacterially expressed GST-fusion proteins was performed using the Glutathione sepharose 4B column (GE Healthcare) under a conventional condition.

### Cell growth analysis

Cell growth was calculated using IncuCyte ZOOM Live Cell Imaging (Essen BioScience, Ann Arbor, MI). The default software parameters for a 6-well plate (Corning, Albany, NY) with a 10× objective were used for imaging. IncuCyte software was used to calculate mean confluence from 121 non-overlapping bright phase images from each well.

### Immunofluorescent staining of cell lines

The cells were semi-fixed in 4% paraformaldeyde for 10 min. After washing in phosphate-buffered saline with Tween 20 (PBST), the cells were fixed in 4% paraformaldehyde for 1 h. Cells were permeabilized in 100% ethanol at −20 °C overnight. After washing in PBST, the cells were incubated with 10% milk for 1 h. The cells were then incubated with 1:100 diluted anti-HER2/ErbB2 rabbit antibody (#4290. Cell Signaling Technology) or anti-cytokeratin 19 mouse antibody (sc-6278. Santa Cruz Biotechnology) at 4 °C overnight. After washing in PBST, the cells were incubated with 10% milk for 1 h. The cells were incubated with 1:100 diluted Alexa Fluor 594 goat anti-rabbit antibody (A11012. Life Technologies, Carlsbad, CA) or Alexa Fluor 488 goat anti-mouse antibody (A11029. Life Technologies) for 1 h and then incubated in NaN_3_-containing PBST for 10 min three times. Coverslips were mounted using Vectashield mounting medium with DAPI (Vector Laboratories, Burlingame, CA).

### Immunohistochemical analysis of clinical samples

Lung cancer tissues (n = 86) were randomly obtained from the patients who underwent surgery between April and December 2013 at Okayama University Hospital (Okayama, Japan). All study methods were carried out in accordance with the guidelines of the Declaration of Helsinki. All experimental protocols were approved by an Institutional Review Board of Okayama University (Permission No. 2041 and No. 2177). Informed consent were obtained from all the patients. Tissue samples were fixed in 10% formaldehyde and embedded in paraffin. The immunohistochemical staining for KRT19 was performed with Novocastra Cytokeratin 19 primary antibody (PA0799. Leica Biosystems, Newcastle Upon Tyne, UK). The detailed protocol for the immunohistochemical staining has been described previously^36^. Immunohistochemical experiments for HER2 were performed with an automated immunostain (Ventana Medical System, Tuscon, AZ) using PATHWAY anti-HER2-neu (4B5) rabbit monoclonal primary antibody (790-2991. Ventana Medical System).

Assessment of HER2 and KRT19 scoring conformed to the HercepTest score. The details of the scores were classified as follows: 1) Score 0: No staining is observed or membrane staining is observed in less than 10% of the tumor; 2) Score 1+: A very faint membrane staining is detected in >10% of the tumor cells, and the cells are only stained in parts of their membrane; 3) Score 2+: A weak-to-moderate complete staining in >10% of the tumor cells; 4) Score 3+: A strong complete membrane staining is observed in >10% of the tumor cells. Scores 0 and 1+ are assessed as negative, and scores 2+ and 3+ are assessed as positive.

### RNA interference studies

RNA interference of KRT19 was performed using small interfering RNA (siRNA). siRNA was transfected into the cells by electroporation using the Neon transfection system (Invitrogen)^37^. After dissociation, cells were resuspended in 9.5 μl of Neon Resuspension Buffer R for every one million cells. For each electroporation, cells and 0.5 μl of Silencer^®^ Select siRNA KRT19 s7998 (10 μM, Ambion, Austin, TX), Silencer^®^ Select siRNA ErbB2 s611 (10 μM, Ambion) or Silencer^®^ Select Negative Control #2 siRNA (10 μM, Ambion) were aliquoted into a sterile microcentrifuge tube. A neon tip was inserted into the neon pipette, and the cell-siRNA mixture was aspirated into the tip avoiding air bubbles. The neon pipette was then inserted into the neon tube containing 3 ml of neon electrolytic buffer E in the neon pipette station. NCI-H1781 and NCI-H2170 cells were pulsed once with a voltage of 1,400 V and width of 20 ms. After the pulse, cells were quickly transferred into 6-well standard plates (Corning). We repeated the same electroporation and the following cell culture four times for each well.

### Statistical analyses

All statistical analyses in this study were performed using EZR (Saitama Medical Center, Jichi Medical University, Saitama, Japan), which is a graphical user interface for R (The R Foundation for Statistical Computing, Vienna, Austria)^38^. More precisely, it is a modified version of R commander designed to add statistical functions frequently used in biostatistics. *P* < 0.05 was considered statistically significant. The microarray data for the National Cancer Center in Japan^39^ is available at Gene Expression Omnibus (GEO; http://www.ncbi.nlm.nih.gov/geo/; accession number GSE31210). Additionally, transcriptome data for the lung adenocarcinoma patients^40^ is available at the cancer genome atlas (TCGA) data portal (https://tcga-data.nci.nih.gov/tcga/tcgaHome2.jsp). Gene set enrichment analysis (GSEA)^41^ was performed to understand background molecular basis.

## Additional Information

**How to cite this article**: Ohtsuka, T. *et al*. Interaction of cytokeratin 19 head domain and HER2 in the cytoplasm leads to activation of HER2-Erk pathway. *Sci. Rep.* **6**, 39557; doi: 10.1038/srep39557 (2016).

**Publisher's note:** Springer Nature remains neutral with regard to jurisdictional claims in published maps and institutional affiliations.

## Supplementary Material

Supplementary Data
