# Supplementary material for: Interaction of cytokeratin 19 head domain and HER2 in the cytoplasm leads to activation of HER2-Erk pathway
Source: Sci Rep. 2016 Dec 23;6:39557. doi: 10.1038/srep39557 (PMC5180104; doi:10.1038/srep39557)
Supplement: Supplementary Data [file srep39557-s1.pdf]

Interaction of cytokeratin 19 head domain and HER2 in the cytoplasm leads to activation of HER2-Erk pathway

Tomoaki Ohtsuka[1], Masakiyo Sakaguchi[2], Hiromasa Yamamoto[1], Shuta Tomida[3], Katsuyoshi Takata[4], Kazuhiko Shien[1], Shinsuke Hashida[1,5], Tomoko Miyata-Takata[4], Mototsugu Watanabe[1], Ken Suzawa[1], Junichi Soh[1], Chen Youyi[2], Hiroki Sato[1], Kei Namba[1], Hidejiro Torigoe[1], Kazunori Tsukuda[1], Tadashi Yoshino[4], Shinichiro Miyoshi[1] and Shinichi Toyooka[1,5*]

Departments of [1]Thoracic, Breast and Endocrinological Surgery, [2]Cell Biology, [3]Biobank, [4]Pathology and [5]Clinical Genomic Medicine, Okayama University Graduate School of Medicine, Dentistry and Pharmaceutical Sciences, 2-5-1 Shikata-cho, Kita-ku, Okayama 700-8558, Japan

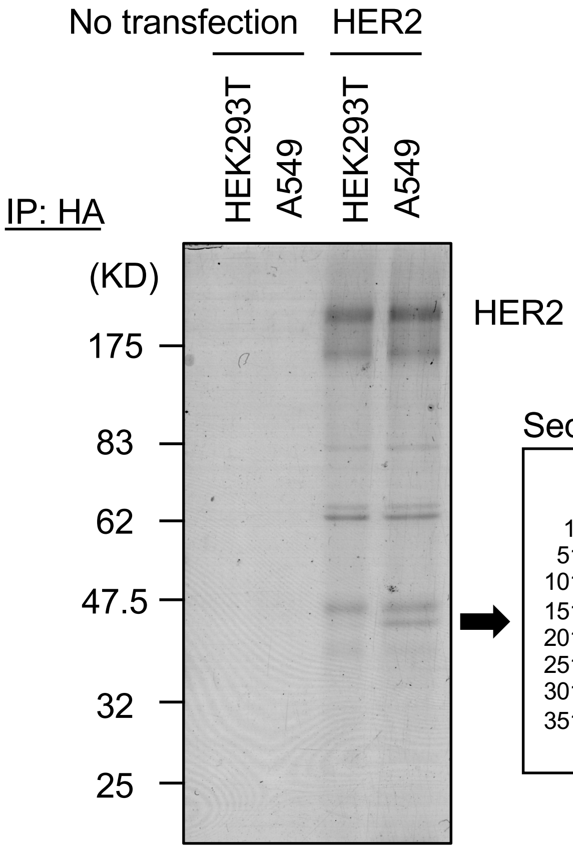

Sequence of the protein binding to HER2

1	MTSYSYR**QSS ATSSFGGLGG GSVRFGPGVA FR**APSIHGGS GGRGVSVSSA
51	RFVSSSSSGA YGGGYGGVLT ASDGLLAGNE K**LTMQNLNDR LASYLDKVR**A
101	LEAANGELEV KIRDWYQKQG **PGPSRDYSHY YTTIQDLRDK** ILGATIENSR
151	**IVLQIDNAR**L AADDFRTKFE TEQALRMSVE ADINGLRR**VL DELTLARTDL**
201	**EMQIEGLKEE LAYLKK**NHEE EISTLRGQVG GQVSVEVDSA PGTDLAKILS
251	DMR**SQYEVMA EQNRKDAEAW FTSRTEELNR EVAGHTEQLQ MSR**SEVTDLR
301	RTLQGLEIEL QSQLSMKAAL EDTLAETEAR FGAQLAHIQA LISGIEAQLG
351	DVRADSERQN QEYQRLMDIK **SRLEQEIATY RSLLEGQEDH YNNLSASK**VL

Supplementary Figure S1

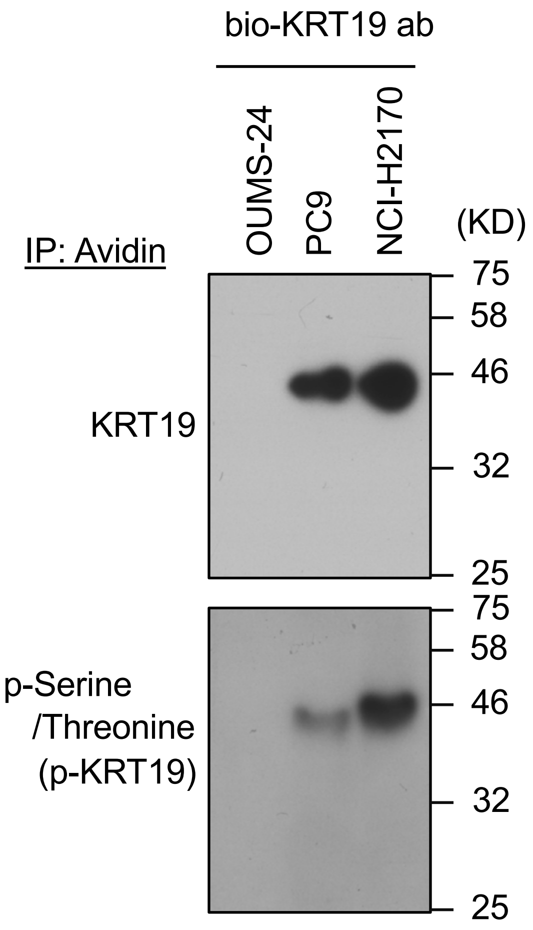

Supplementary Figure S2

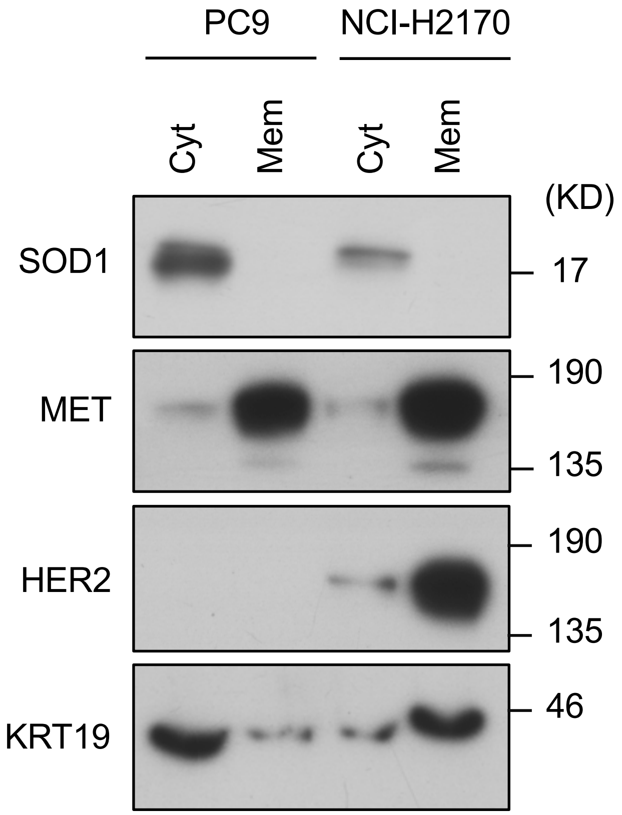

Supplementary Figure S3

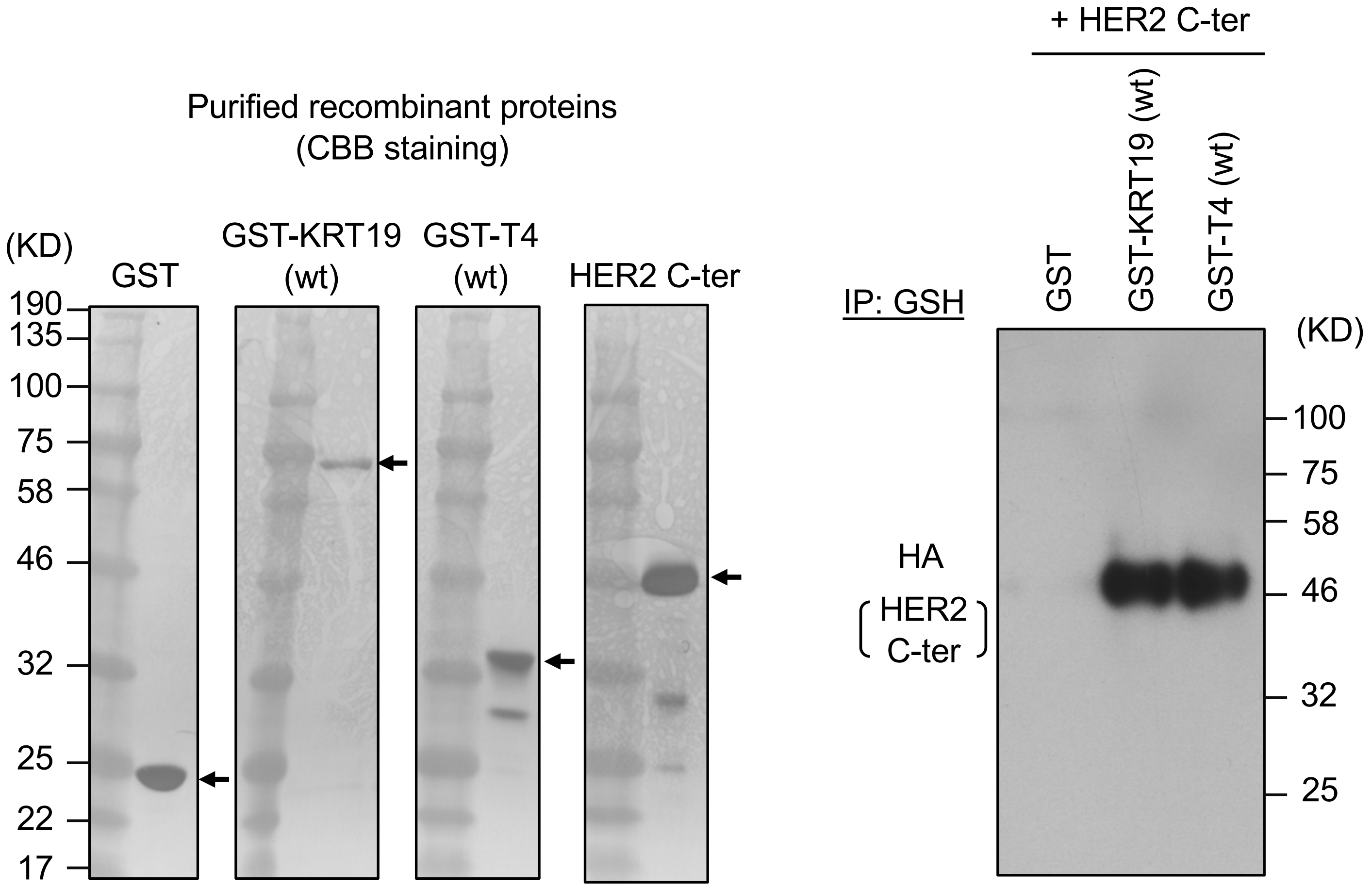

Supplementary Figure S4

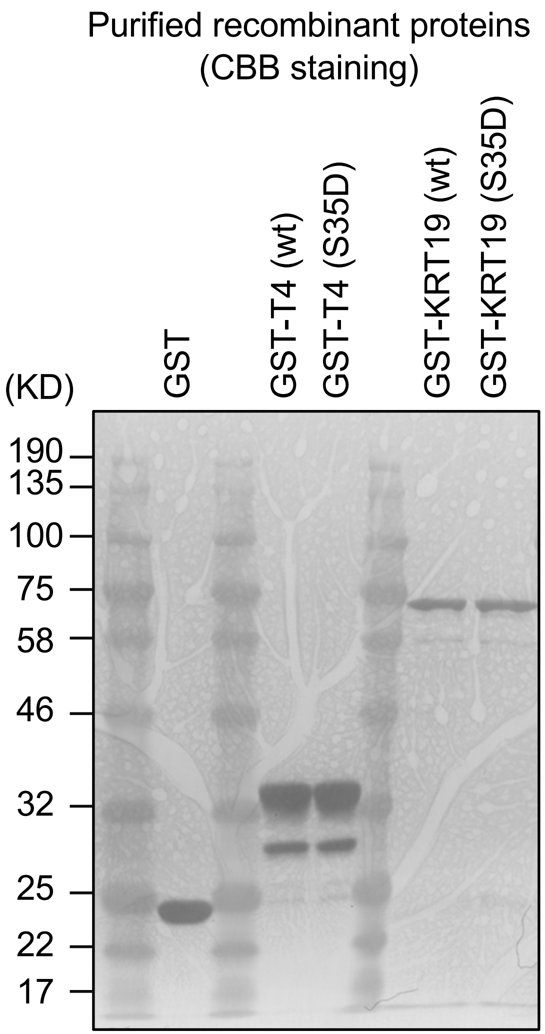

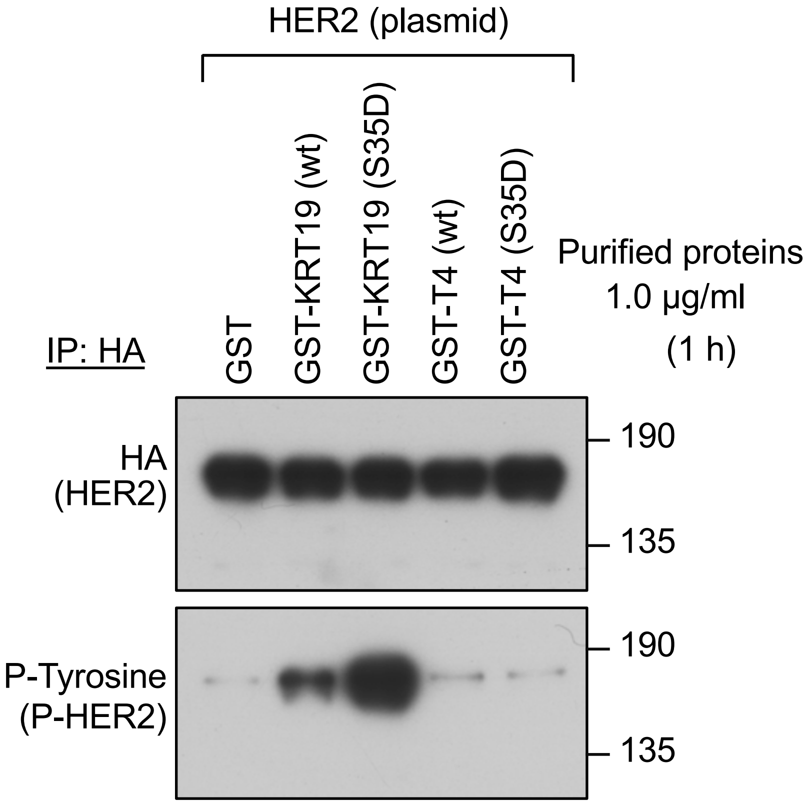

Supplementary Figure S5

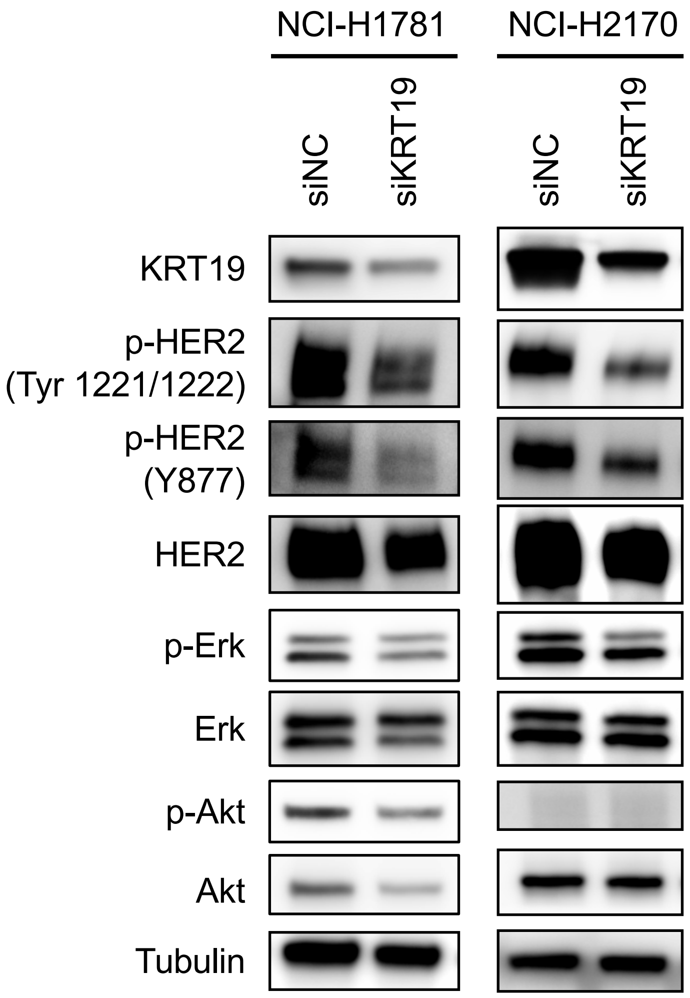

Supplementary Figure S6

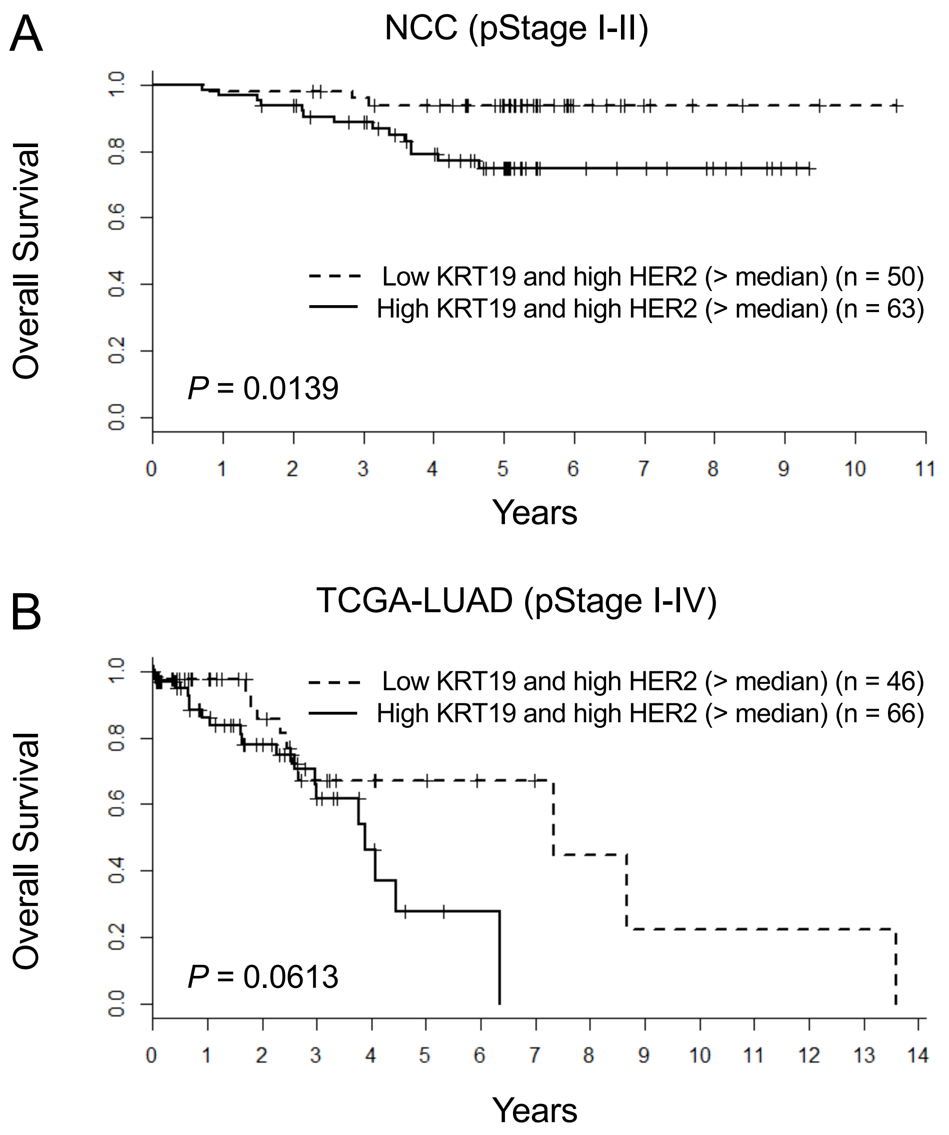

Supplementary Figure S7

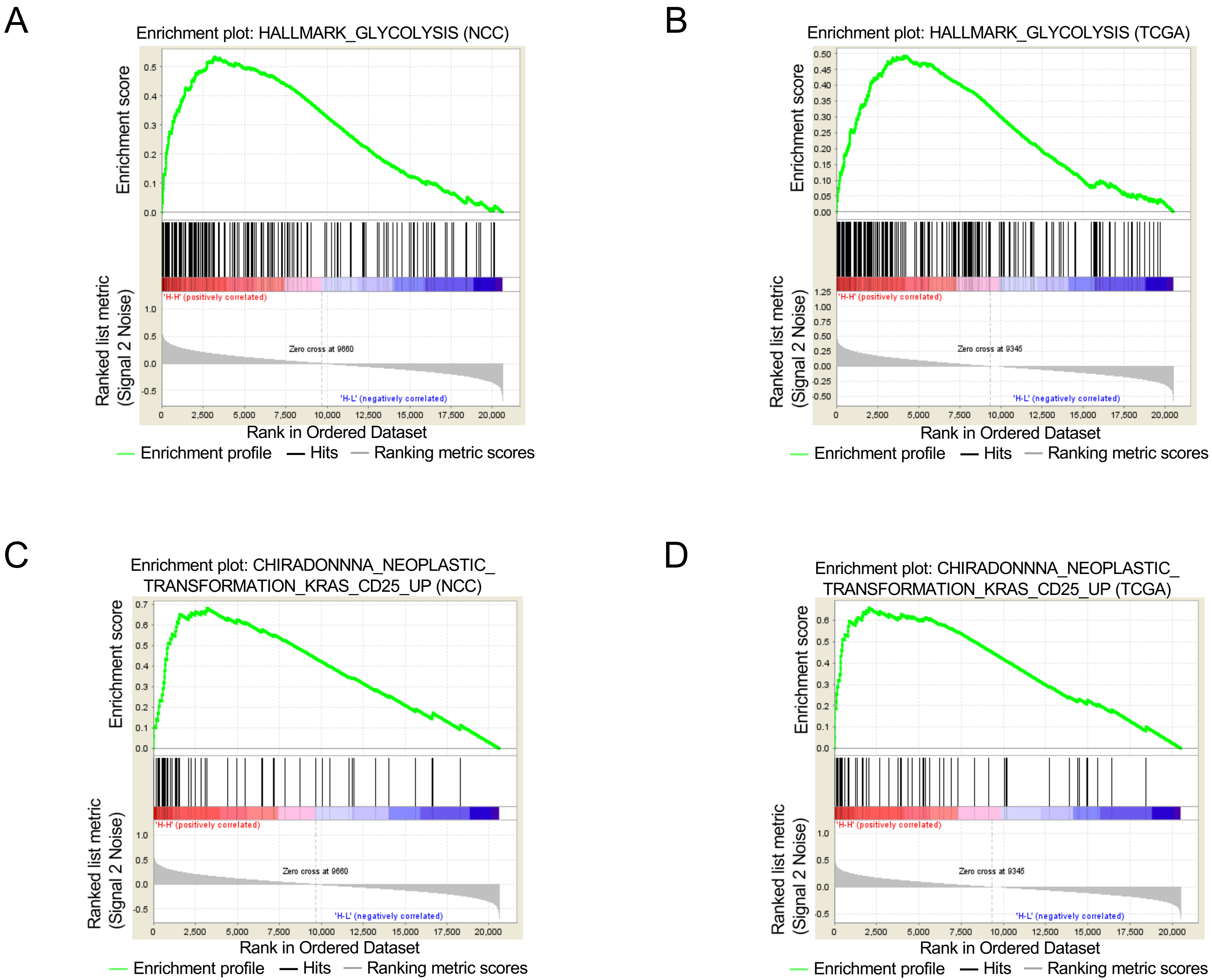

Supplementary Figure S8

Figure 1A

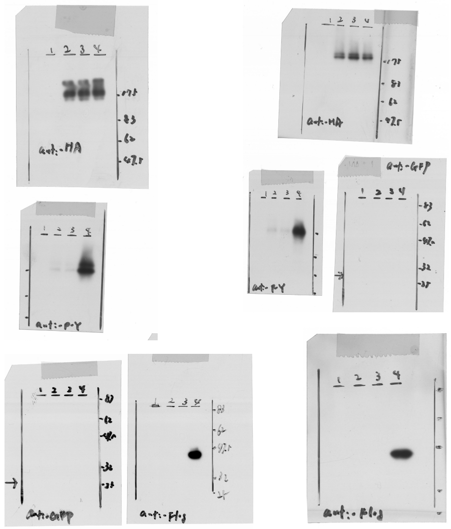

Figure 1B

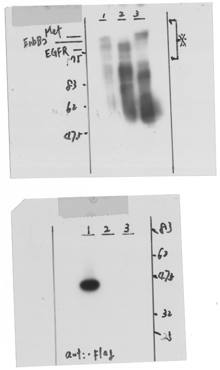

Figure 2A

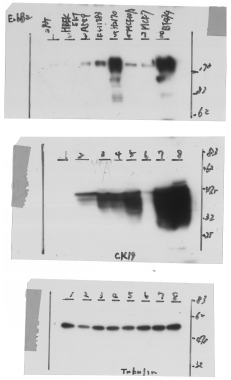

Figure 3B

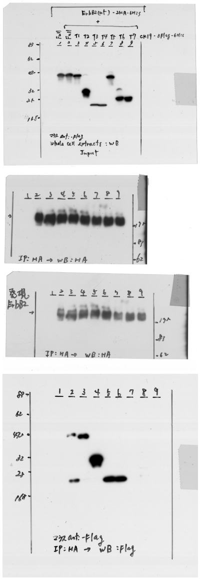

Figure 3D

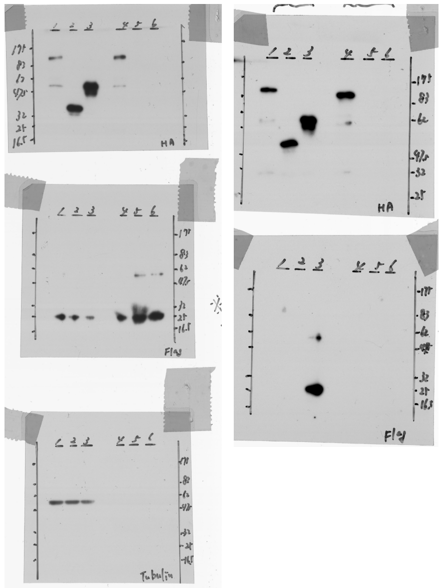

Figure 5

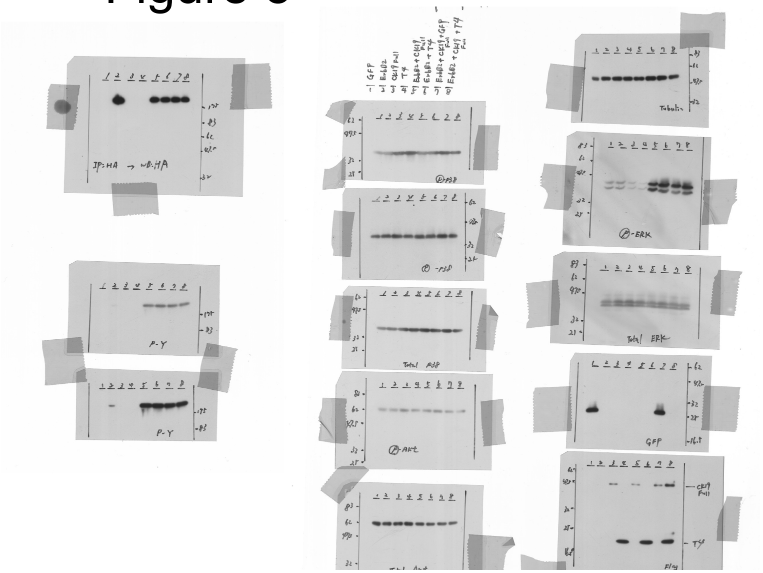

Figure 6A

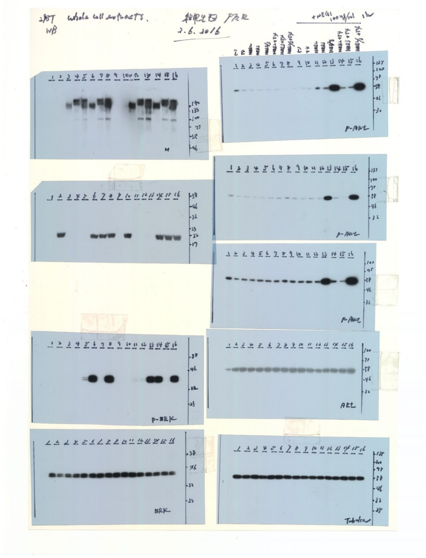

Figure 6B

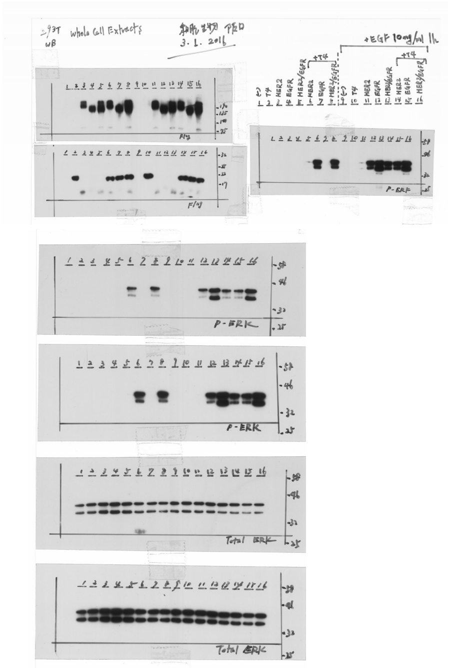

Supplementary Figure S9

Figure 4A

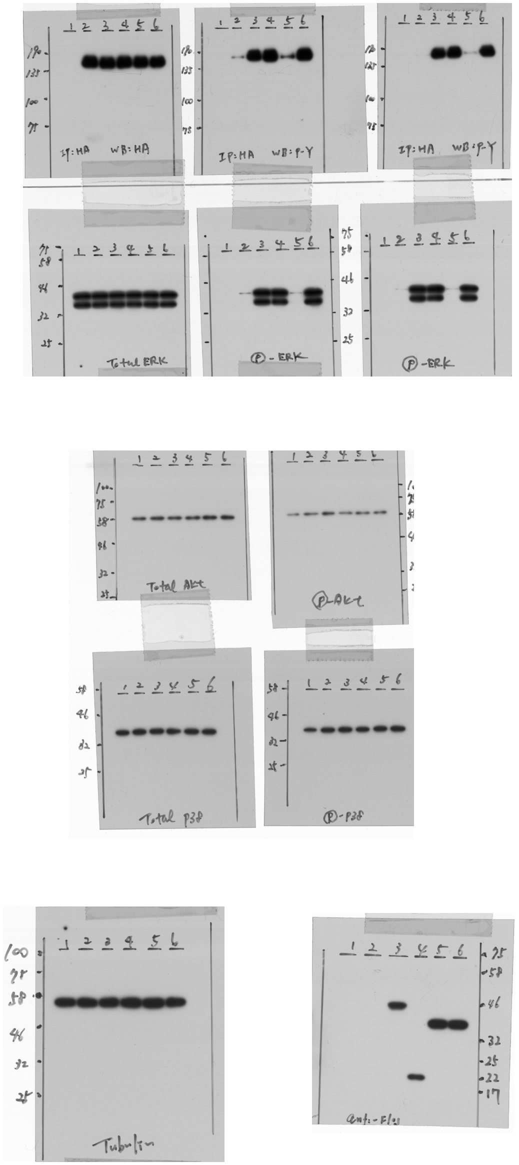

Figure 4B

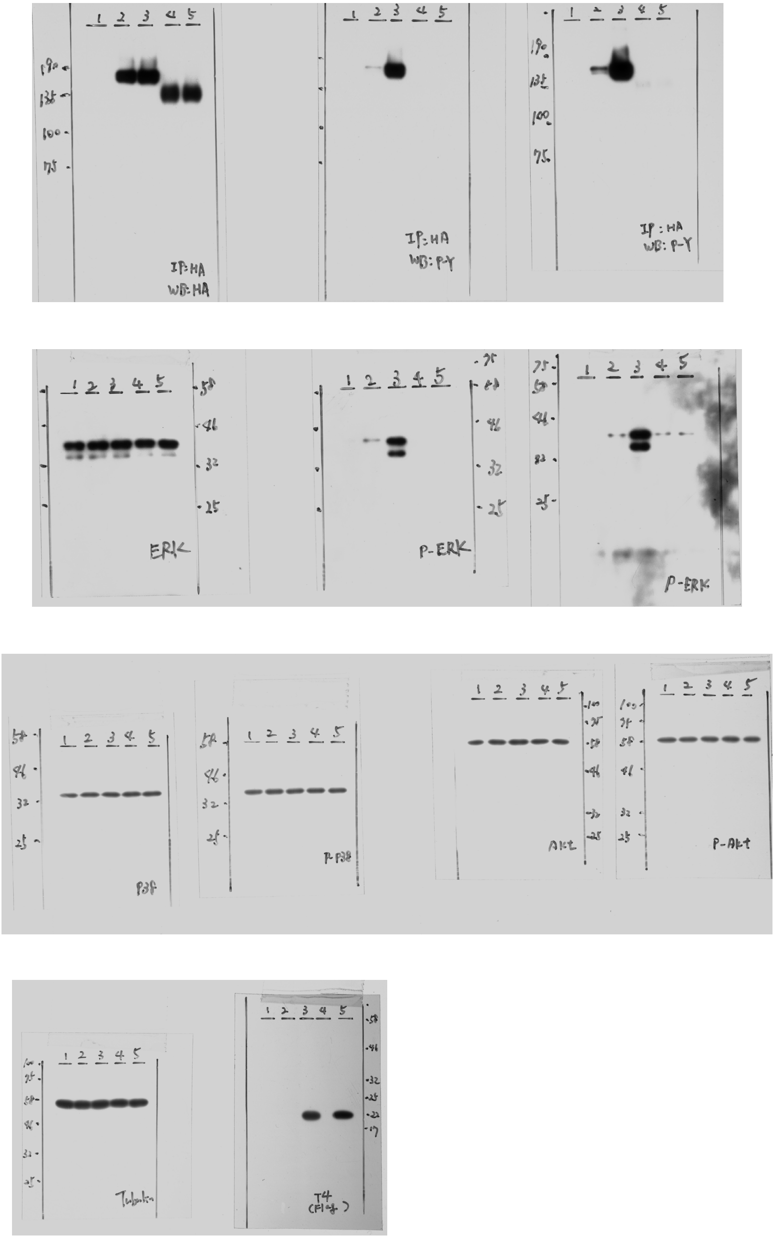

Supplementary Figure S10

Supplementary Figure legends

Supplementary Figure S1 KRT19 was detected as a protein binding to HER2. HER2 was transfected to HEK293T and the non-small cell lung cancer cell line A549. Next, HER2 complexes were collected using anti-HA-tag immunoprecipitation. Those samples were electrophoresed and silver-stained. The protein band observed only in A549 was examined by LC–Ms/Ms analysis. Matched peptides were shown as bold and underlined.

Supplementary Figure S2 Phosphorylation profiles of endogenous KRT19. OUMS-24, PC-9 and NCI-H2170 cells were lysed and subjected to immunoprecipitation using the KRT19 biotinylated antibody and following Western blotting with either anti-KRT19 antibody or with anti-phospho-Serine/Threonine antibody.

Supplementary Figure S3 Cell fractionation analysis of HER2 and KRT19. PC-9 and NCI-H2170 cells were fractionated to cytosol and membrane. Anti-SOD1 antibody was used to confirm proper isolation of cytosolic fraction. Anti-MET antibody was used to assess the successful enrichment of membrane fraction.

Supplementary Figure S4 Recombinant GST, GST-KRT19 (wt) and GST-T4 (wt) were purified from an *E. Coli* expression system. Recombinant HER2-C terminal domain-3HA-6His (HER2 C-ter) was purified from HEK293T expression system. The purified proteins were subjected to SDS-PAGE and detected by Coomassie Brilliant Blue (CBB) staining. *In vitro* binding analysis was performed to assess direct interaction for each of the purified proteins (GST, GST-KRT19 (wt) and GST-T4 (wt))

to the purified HER2-C-ter protein. The GST proteins was incubated with HER2-C ter protein, precipitated by Glutathione Sepharose 4B beads (GSH beads), and analyzed by Western blotting for the precipitates.

Supplementary Figure S5 Recombinant GST, GST-T4 (wt and S35D) and GST-KRT19 (wt and S35D) were purified from an *E. Coli* expression system. The purified proteins were subjected to SDS-PAGE and detected by Coomassie Brilliant Blue (CBB) staining. After HER2 was transfected to HEK293T, purified GST proteins were administered to the cultures.

Supplementary Figure S6

The KRT19 in NCI-H1781 and NCI-H2170 were suppressed using siRNA. Knockdown of KRT19 resulted in the inhibition of phospho- Erk. siNC, negative control of siRNA.

Supplementary Figure S7 Impact of *KRT19* and *HER2* mRNA expressions on prognosis of lung cancer patients. (A) Overall survival in the patients with high *HER2* expression was significantly worse in the cases with high expression of KRT19 in National Cancer Center in Japan (NCC) dataset. (B) Similar trend was observed in the cancer genome atlas (TCGA) dataset, although the significance was marginal.

Supplementary Figure S8 Gene set enrichment analysis (GSEA) was performed using the 2 datasets from National Cancer Center in Japan (NCC) and the cancer genome atlas (TCGA). (A, B) Among the gene sets tested, “HALLMARK_GLYCOLYSIS” was most enriched in both datasets. (C, D) Ras-related gene set was significantly enriched in both datasets with top rank in total

score (See Supplementary Table S2).

Supplementary Figure S9, S10 Full-length blots in the main paper are presented.

Supplementary Table S1 Correlations between HER2 and KRT19 expression in surgically resected non-small cell lung cancer.

		HER2 expression status		*P* value
		(+)	(-)	
KRT19 expression status (n=86)	Positive	36	34	0.001
	Negative	1	15	
KRT19 location in KRT19 (+) cases (n=70)	Cell membrane	34	23	0.0052
	Cytoplasm	2	11	

Supplementary Table S2 Ranking of gene sets in NCC and TCGA datasets

Total rank	NAME	Rank in NCC datasets	Rank in TCGA datasets	Total score of the rank in both datasets
1	CHIARADONNA_NEOPLASTIC_TRANSFORMATION_KRAS_CDC25_UP	3	4	7
2	GRUETZMANN_PANCREATIC_CANCER_UP	2	10	12
3	HUPER_BREAST_BASAL_VS_LUMINAL_DN	16	1	17
4	LI_LUNG_CANCER	19	2	21
5	ZHONG_SECRETOME_OF_LUNG_CANCER_AND_FIBROBLAST	1	35	36
6	RICKMAN_TUMOR_DIFFERENTIATED_WELL_VS_POORLY_DN	33	9	42
7	REN_ALVEOLAR_RHABDOMYOSARCOMA_DN	4	39	43
8	PROVENZANI_METASTASIS_DN	10	33	43
9	LEI_MYB_TARGETS	6	38	44

10	WU_CELL_MIGRATION	37	20	57
11	CHEN_LUNG_CANCER_SURVIVAL	5	62	67
12	BRUECKNER_TARGETS_OF_MIRLET7A3_DN	22	47	69
13	REACTOME_ANTIGEN_PROCESSING_CROSS_PRESENTATION	17	55	72
14	BIOCARTA_CDC42RAC_PATHWAY	29	54	83
15	ENK_UV_RESPONSE_EPIDERMIS_UP	59	25	84
16	VANTVEER_BREAST_CANCER_ESR1_DN	69	24	93
17	RICKMAN_TUMOR_DIFFERENTIATED_MODERATELY_VS_POORLY_UP	21	89	110
18	HUMMERICH_SKIN_CANCER_PROGRESSION_UP	28	83	111
19	WINTER_HYPOXIA_UP	56	60	116
20	HONMA_DOCETAXEL_RESISTANCE	61	59	120
21	DELYS_THYROID_CANCER_UP	44	78	122
22	ZHANG_PROLIFERATING_VS_QUIESCENT	80	44	124
23	MACLACHLAN_BRCA1_TARGETS_UP	85	41	126
24	ZHONG_SECRETOME_OF_LUNG_CANCER_AND_ENDOTHELIUM	68	63	131
25	KOHOUTEK_CCNT1_TARGETS	126	11	137
26	JISON_SICKLE_CELL_DISEASE_UP	137	3	140
27	SHIPP_DLBCL_VS_FOLLICULAR	23	118	141

	_LYMPHOMA_UP			
28	AMIT_SERUM_RESPONSE_240_MCF10A	104	42	146
29	LIN_APC_TARGETS	110	40	150
30	ONDER_CDH1_TARGETS_2_DN	81	71	152

NCC, National Cancer Center in Japan; TCGA, the cancer genome atlas
